# A188 ENHANCED MICROBIAL TRYPTOPHAN METABOLISM ACTIVATES THE ARYL HYDROCARBON RECEPTOR AND AMELIORATES INTESTINAL INFLAMMATION

**DOI:** 10.1093/jcag/gwad061.188

**Published:** 2024-02-14

**Authors:** L Rondeau, B Barbosa, R Dang, A Caminero

**Affiliations:** Medicine, McMaster University, Oakville, ON, Canada; Medicine, McMaster University, Oakville, ON, Canada; Medicine, McMaster University, Oakville, ON, Canada; Medicine, McMaster University, Oakville, ON, Canada

## Abstract

**Background:**

Intestinal microbiota, diet, and the immune system have been proposed to contribute to the development of inflammatory bowel diseases (IBD). The aryl hydrocarbon receptor (AhR) is a critical regulator of intestinal immunity and mucosal barrier homeostasis that is activated by agonists such as host and microbial tryptophan metabolites. IBD patients have altered microbiota and reduced AhR agonists in intestinal content resulting in downregulation of AhR. Our recent findings indicate that mice harbouring microbiota with impaired tryptophan metabolism and AhR agonist production develop more severe inflammation during colitis.

**Aims:**

To study the influence of diet-microbe interventions on AhR activation and intestinal inflammation in mice with impaired microbial tryptophan metabolism.

**Methods:**

Germ-free C57BL/6 mice from McMaster’s Axenic Gnotobiotic Unit were colonized with cecal content of mice harbouring reduced microbiota with impaired tryptophan metabolism with and without tryptophan metabolizing *Clostridium sporogenes*. Following three weeks of microbial stabilization, mice were provided a high tryptophan (HT) diet or a low tryptophan (LT) diet as a control. Experimental colitis was induced using dextran sulfate sodium in drinking water (DSS; 2.5% w/v). Briefly, DSS was provided *ad libitum* for five days, followed by two days of recovery before tissue collection. Activation of AhR was measured in stool and *C. sporogenes* cultures using an *in vitro* AhR luciferase reporter assay. AhR agonists were quantified by capillary-electrophoresis-mass spectrometry. Colonic expression of AhR pathway genes was evaluated by RT-qPCR. Susceptibility to colitis was assessed by histologic analysis of distal colon sections, fecal lipocalin-2, weight loss, and clinical scores. Inflammatory gene transcripts were quantified in colon tissue by Nanostring.

**Results:**

*C. sporogenes* efficiently produces AhR agonists *in vitro* and *in vivo*. In mice with impaired tryptophan metabolism, the HT diet increased AhR agonists in intestinal content and colonic AhR pathway gene expression, which was further enhanced in mice colonized with *C. sporogenes*. Compared to mice fed LT and HT diets, mice colonized with *C. sporogenes* and fed HT were most protected from weight loss, tissue damage, and immune cell infiltration.

**Conclusions:**

These findings highlight the potential of tryptophan-rich diets and targeted microbe interventions as a novel approach to enhance AhR activation and mitigate inflammation during colitis. Consequently, exploring methods to improve tryptophan metabolism through diet and manipulation of the microbiota could be a promising therapeutic avenue for IBD patients with impaired AhR activation.

**Funding Agencies:**

CCC, CIHR

